# Octafluoro-Substituted Phthalocyanines of Zinc, Cobalt, and Vanadyl: Single Crystal Structure, Spectral Study and Oriented Thin Films

**DOI:** 10.3390/ijms24032034

**Published:** 2023-01-19

**Authors:** Aleksandr Sukhikh, Darya Klyamer, Dmitry Bonegardt, Tamara Basova

**Affiliations:** Nikolaev Institute of Inorganic Chemistry SB RAS, 3 Lavrentiev Pr., 630090 Novosibirsk, Russia

**Keywords:** metal phthalocyanines, fluorine substituents, single crystal structure, thin films, X-ray diffraction, conductivity

## Abstract

In this work, octafluoro-substituted phthalocyanines of zinc, vanadyl, and cobalt (MPcF_8_, M = Zn(II), Co(II), VO) were synthesized and studied. The structures of single crystals of the obtained phthalocyanines were determined. To visualize and compare intermolecular contacts in MPcF_8_, an analysis of Hirshfeld surfaces (HS) was performed. MPcF_8_ nanoscale thickness films were deposited by organic molecular beam deposition technique and their structure and orientation were studied using X-ray diffraction. Comparison of X-ray diffraction patterns of thin films with the calculated diffractograms showed that all three films consisted of a single crystal phase, which corresponded to a phase of single crystals. Only one strong diffraction peak corresponding to the plane (001) was observed on the diffraction pattern of each film, which indicated a strong preferred orientation with the vast majority of crystallites oriented with a (001) crystallographic plane parallel to the substrate surface. The effect of the central metals on the electronic absorption and vibrational spectra of the studied phthalocyanines as well as on the electrical conductivity of their films is also discussed.

## 1. Introduction

Phthalocyanines (MPc) have been known for more than a hundred years but are still of wide interest to researchers not only as dyes and pigments but also as molecular semiconductors, catalysts, and photosensitizers. Due to the extensive π-system and the ability to form supramolecular ensembles in the form of columns of parallel packed molecules, they have a number of important and interesting properties. Today they are used in various branches of science and technology, such as chemistry [1,2], physics [3,4], and medicine [5,6,7].

An important advantage of phthalocyanines is the ability to vary their structure widely by replacing the central metal and substituents, both in the aromatic macrocycle and in axial positions. Such modification leads to a significant change in the solubility of phthalocyanine derivatives, as well as their optical, electrophysical, and catalytic properties [8,9,10,11,12]. For example, the introduction of fluorine substituents into the phthalocyanine ring leads to a decrease in their solubility in conventional organic solvents, but fluorinated phthalocyanines become capable of transferring into the gaseous phase without decomposition in a vacuum [13,14], which makes it possible to obtain their homogeneous thin films using a physical vapor deposition method. It has been shown that the introduction of fluorine substituents leads to a change in conductivity and transport properties [15]. For example, unlike ZnPc, which exhibits *p*-type semiconductor behavior, ZnPcF_8_ and ZnPcF_16_ are *n*-type semiconductors, while ZnPcF_4_ can demonstrate ambipolar behavior. Due to these electrical properties, fluorinated metal phthalocyanines are widely used as active layers of diodes and organic field-effect transistors (OFET) [16,17,18]. Another important area of their application is the active layers of chemical sensors for the detection of hazardous gases and substances in aquatic environments [19,20,21]. To use phthalocyanines in electronic devices, it is important to obtain thin films with a controlled structure and ordering on various surfaces. The study of the structural features of their single crystals and the packing of molecules in thin films can reveal the influence of π–π interactions and other molecular contacts on the properties of phthalocyanine-based materials. It is also worth noting that the structure and phase composition of phthalocyanine films of nanoscale thickness may differ from the structure of their single crystals and powders [22,23,24]; for this reason, special attention should be paid to the study of the structural features of thin films since they determine the electrical and sensor properties.

Among fluoro-substituted metal phthalocyanines, hexadecafluoro-substituted derivatives are the most studied. The structure of single crystals of MPcF_16_ (M=Cu, Co, Zn, Pd, Fe, Pb, VO) [22,25,26] and the structural features of their films [27,28] have already been reported in a number of publications. The effect of morphology and ordering in MPcF_16_ films on their electrical properties and OFET characteristics has also been discussed by many researchers [29,30,31]. Recently we studied structures of single crystals and thin films of a series of tetrafluoro-substituted phthalocyanines bearing F-substituents both in the peripheral (MPcF_4_-p, M=Co, Cu, Zn, Pd, Fe, Pb, VO) [13,32] and non-peripheral (MPcF_4_-np, M= Co, Cu, Zn, Pd, Fe, Pb, VO) [23,33] positions in the phthalocyanine macroring. Similar to MPcF_16_, MPcF_4_ films are also widely studied as active layers of OFETs [34] and chemical sensors [33]. It has been shown that the introduction of different numbers of F-substituents leads to alteration of the intermolecular contacts and as a result a change in the crystal structure and packing of the phthalocyanine molecules. This also affects their thermodynamic [13] and electrical [35,36] properties as well as sensor performance [28]. At the same time, octafluoro-substituted metal phthalocyanines have not been studied in detail and work on the study of the structure of their single crystals and films have been sporadic. For example, Jiang et al. [37] refined the structures of ZnPcF_8_ and CuPcF_8_ single crystals and studied charge-carrier mobility in their single crystals. The structures of anionic salts of CuPcF_8_ with tetrabutyl ammonium and (triphenylphosphoranylidene) ammonium were also investigated [38]. Brinkmann et al. [35] studied vapor-deposited ZnPcF_8_ films using UV-vis absorption spectroscopy and scanning electron microscopy and investigated change in their current during exposure to oxygen. Several works have been devoted to the study of molecule–substrate interface states in ultrathin ZnPcF_8_ films deposited onto single-crystal substrates using scanning tunneling microscopy and photoemission spectroscopy [39,40]. At the same time, crystals and films of other MPcF_8_ derivatives have not yet been studied.

To fill this gap, octafluoro-substituted phthalocyanines of zinc, vanadyl, and cobalt (MPcF_8_, M=Zn(II), Co(II), VO) were synthesized and studied in this work. The structures of single crystals of the obtained phthalocyanines were determined. To visualize and compare intermolecular contacts in MPcF_8_, an analysis of Hirshfeld surfaces (HS) was performed. MPcF_8_ nanoscale thickness films were deposited using the organic molecular beam deposition (OMBD) technique and their structure and orientation were studied using X-ray diffraction. The effect of the central metals on the electronic absorption and vibrational spectra of the studied phthalocyanines as well as on the electrical conductivity of their films is also discussed.

## 2. Results and Discussion

### 2.1. MPcF_8_ Crystal Structures

Single crystals of ZnPcF_8_, CoPcF_8_, and VOPcF_8_ were obtained as a result of sublimation in a vacuum (10^−2^ Torr). ZnPcF_8_ crystals form as relatively large clusters up to 1 mm in length and 60 μm in width. CoPcF_8_ crystals grow in the form of thin needles or long ribbons (up to 300 μm long, 30 microns wide, and less than 10 microns thick), with a tendency to bend and split into several smaller crystals. VOPcF_8_ crystals have the form of small (<100 μm wide and <30 μm thick) elongated hexagonal plates. All crystals have a dark purple color and a metallic luster typical of phthalocyanines. Unit cell parameters and refinement statistics for MPcF_8_ are given in Table 1. The main intermolecular and interatomic distances in MPcF_8_ are summarized in Table 2.

**Figure 1:**
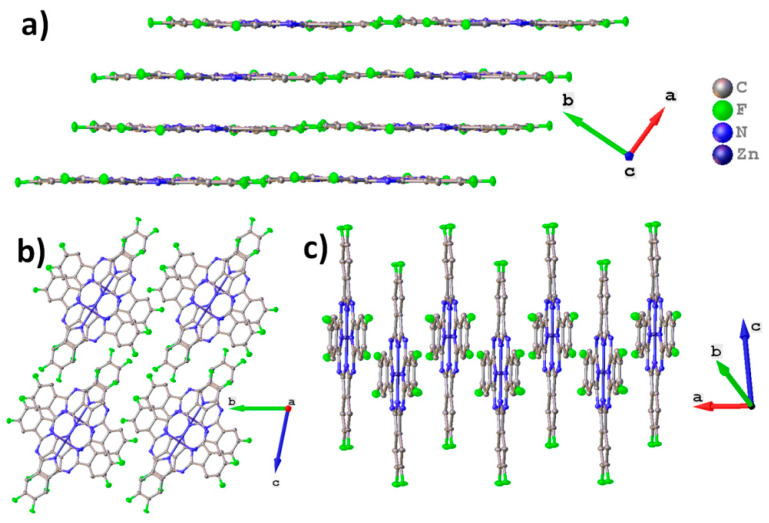
Molecular packing diagrams for ZnPcF_8_: molecular layers along the *c* axis (**a**), stacks along the *a* axis (**b**), and arrangement of molecules in an individual stack (**c**).

Figure 1 shows the molecular packing for ZnPcF_8_. ZnPcF_8_ crystallizes in a triclinic system with Z=2, which is identical to the ZnPcF_8_ crystal structure previously reported by Jiang et al. [37]. ZnPcF_8_ molecules are arranged in layers along the *c* axis (Figure 1a). This is typical for planar phthalocyanines, which usually crystallize in uniform stacks or in a “herringbone” pattern. The distance between layers is 3.173 Å and between the planes of adjacent molecules is 3.217 Å; the angle between the layer and the individual molecule (planes constructed through all atoms except hydrogen) is only 0.66°. When viewed along the *a* axis (Figure 1b), it seems that ZnPcF_8_ molecules form stacks, but the molecules inside the stack are staggered, so that the central metals do not lie on the same line as in other planar phthalocyanines (Figure 1c) [13].

**Figure 2:**
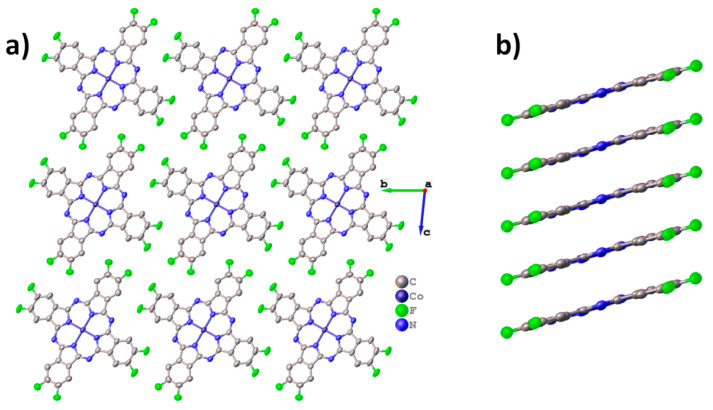
Molecular packing diagrams for CoPcF_8_: molecular stacks along the *a* axis (**a**), molecular arrangement inside the individual stack (**b**).

Molecular packing diagrams for CoPcF_8_ are shown in Figure 2. CoPcF_8_ crystallizes in a triclinic system with Z=1 and is isostructural to the previously studied CuPcF_8_ [15,37], as well as peripherally substituted tetrafluorinated cobalt phthalocyanine (CoPcF_4_-p) [13] and unsubstituted α-CoPc [41].

CoPcF_8_ molecules are packed in uniform stacks along the *a* axis (Figure 2a) with a distance of 3.299 Å between adjacent molecules inside the stack and a stacking angle of 23.81° (the angle between the normal to the molecule plane and the stacking axis). For comparison, these values are 3.318Å and 24.70° in CoPcF_4_-p and 3.414 Å and 24.58° in α-CoPc. The molecular arrangement inside the individual stack is shown in Figure 2b.

**Figure 3:**
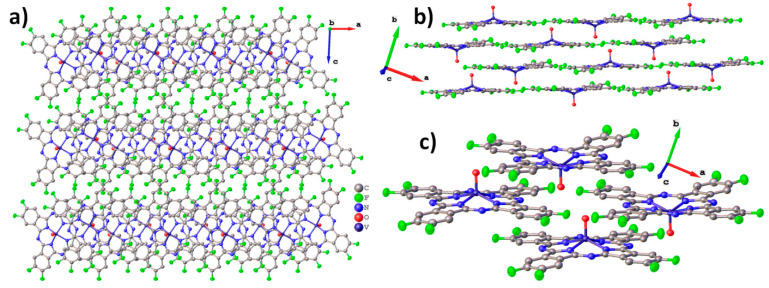
Molecular packing diagrams for VOPcF_8_: the packing motif along the *a* axis (**a**), the arrangement of molecules inside an individual layer (**b**), and a close-up view of the arrangement of neighboring molecules inside the layer (**c**).

Unlike zinc and cobalt phthalocyanines, vanadyl phthalocyanine molecules are not planar, resulting in a different packing style. For instance, the triclinic polymorph of VOPc and VOPcF_4_-p form molecular chains, in which one “facing up” molecule is adjacent to two “facing down” molecules. At the same time, VOPcF_16_ and VOPcF_4_-np form molecular layers, where one “facing up” molecule is adjacent to four “facing down” molecules [13,23,42]. Molecular packing diagrams for VOPcF_8_ are shown in Figure 3. As can be seen when viewed along the *b* axis, VOPcF_8_ molecules do not form continuous 2D layers, since the only type of interaction between molecules along the *c* axis is close F…F and F…H contacts (Figure 3a). Instead, VOPcF_8_ molecules are arranged along the *a* axis with alternating “up” and “down” configurations (Figure 3b,c). The gaps between the molecules in the chain are filled with two oxygen atoms belonging to molecules from two neighboring chains; the distance between these oxygen atoms is 3.300 Å. This molecular arrangement differs significantly from other fluorinated vanadyl phthalocyanines, where the gap between two molecules (chain packing) or four molecules (layered packing) is occupied by only one oxygen atom.

### 2.2. Hirshfeld Surface Analysis

**Figure 4:**
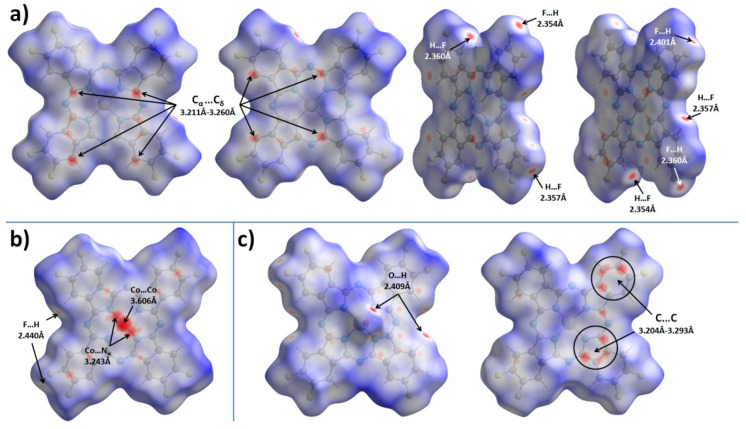
Hirshfeld surfaces for ZnPcF_8_ (**a**), CoPcF_8_ (**b**), and VOPcF_8_ (**c**), mapped with the *d_norm_* property in the range of −0.2–1.45.

To better analyze and visualize intermolecular interactions in MPcF_8_, Hirshfeld surfaces (HS) generated in CrystalExplorer 21.5 [43] and mapped with *d_norm_* (normalized contact distance) and shape index properties, were used. Figure 4 shows HS for MPcF_8_ molecules mapped with the *d_norm_* property. By default, CrystalExplorer selects an individual *d_norm_* scale range for each Hirshfeld surface based on the minimum/maximum values for the specified surface. These values are −0.1495/1.3175 for ZnPcF_8_, −0.1985/1.3833 for CoPcF_8_, and -0.1477/1.4484 for VOPcF_8_, which means that the crystal structure of CoPcF_8_ contains stronger close contacts compared to others. Thus, an arbitrary range of −0.2−1.45 was chosen for the convenience of visual comparison between all three MPcF_8_ compounds. The front and back sides of the HS of ZnPcF_8_ have four red spots arranged in a square shape, which correspond to C_α_…C_δ_ close contacts between neighboring molecules in the range of 3.211 Å−3.260 Å (mean value 3.235 Å). Close H…F and F…H contacts with the shortest distance of 2.354 Å are also well visualized at the edges of the molecules (Figure 4a).

Figure 4b shows only one side of CoPcF_8_ HS, since the crystal structure of CoPcF_8_ contains a single centrosymmetric molecule, which means that the front and back sides of CoPcF_8_ HS are identical. It contains several red spots, which correspond to various close contacts between carbon atoms of neighboring molecules in the stack. These short contacts are much less noticeable than in the case of ZnPcF_8_ since CoPcF_8_ molecules are located 0.087 Å further apart than in ZnPcF_8_. Instead, CoPcF_8_ HS is dominated by three large red spots in the center, which correspond to Co…Co close contact with a distance of 3.606 Å and two reciprocal Co…N_α_ close contacts (3.243 Å). The presence of these close contacts is due to the tendency of the cobalt cation to have octahedral coordination. The angle between the close Co...N_α_ contact and the normal to the square formed by the four N_α_ atoms of the CoPcF_8_ molecule is 174.31°, which is close to 180° for an ideal octahedron. The distance of Co-N_α_ in the molecule is 1.915 Å, while the distance between Co and N_α_ of the adjacent molecule in the stack is 3.243 Å. The side of CoPcF_8_ HS is characterized by four weak red dots, which correspond to four close F...H contacts of 2.440 Å. Although CoPcF_8_ is isostructural with CoPcF_4_-p [13], the contacts between the molecules in their adjacent stacks are different. There are four red spots that correspond to close contacts F…H on HS of both CoPcF_4_-p and CoPcF_8_, but these contacts in CoPcF_4_-p are shorter by 0.139 Å than in the case of CoPcF_8_.

There are two prominent red spots, which correspond to a reciprocal pair of close O…H contacts (2.409 Å), and several dull red spots, which correspond to various C…C contacts and one H…F contact (2.404 Å) on the front side of VOPcF_8_ HS (Figure 4c). Similar O…H close contacts were also observed in the case of the triclinic-II polymorph of VOPc (2.431 Å) [42] and VOPcF_4_-p (2.441 Å) [13]. The back side of VOPcF_8_ HS contains two clusters of red dots, which correspond to close contacts between carbon atoms in the pyrrole fragment of one molecule and carbon atoms in the benzene fragment of a neighboring one (3.204–3.293 Å, mean value 3.254 Å). Despite the large number of F-substituents, the F…H and F…F interactions are less pronounced than in VOPcF_4_-p [13], which is characterized by very strong F…F interactions with the closest distance between fluorine atoms of only 2.281 Å.

**Figure 5:**
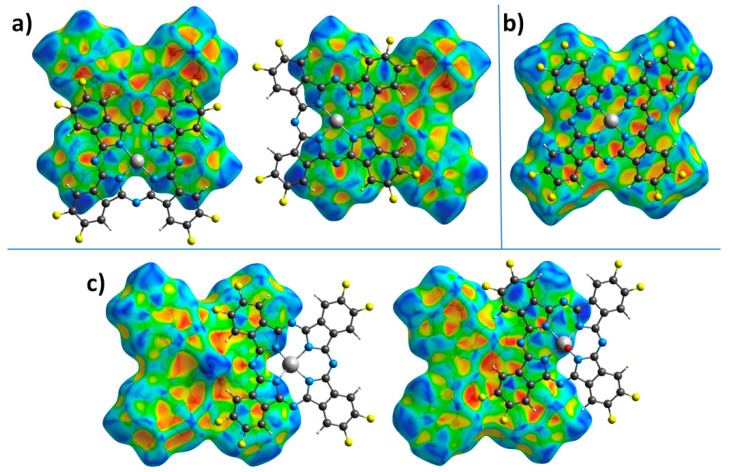
Hirshfeld surfaces for ZnPcF_8_ (**a**), CoPcF_8_ (**b**), and VOPcF_8_ (**c**) molecules, mapped with the shape index property.

Figure 5 shows HS mapped with the shape index property, which is very sensitive to small changes in surface curvature, especially for flat areas with a small overall curvature. In particular, this allows for identifying weak π–π interactions between phthalocyanine molecules, which are displayed on HS in the form of pairs of blue and red triangles arranged in the form of an hourglass. Neighboring molecules are drawn on top of HS for better visualization of π–π interactions.

The front side of ZnPcF_8_ HS shows π–π interactions between two pairs of pyrrole moieties. The back side also has these interactions, but it is rotated by 90° relative to the front surface. This means that among four pyrrole moieties in each ZnPcF_8_ molecule, one fragment participates in two π–π interactions, two pyrrole fragments are involved in one π–π interaction each, and the last pyrrole fragment does not participate in any π–π interactions. The angle/distance/shift values for the π–π interactions between pyrrole fragments in ZnPcF_8_ are 2.603°/3.232 Å/0.777 Å and 1.219°/3.230 Å/0.786 Å.

CoPcF_8_ HS mapped with the shape index property has pairs of red and blue triangles in each benzene and pyrrole moiety, as well as in each of the four segments of the central macrocycle, which means that the whole CoPcF_8_ molecule is involved in π–π interaction with neighboring molecules in the stack. The same character of interactions is also observed in α-CoPc [41] and CoPcF_4_-p [13]. The angle values for π–π interactions are 0° for each fragment, while distance and shift are in the range of 3.251–3.357 Å and 1.318–1.561 Å, respectively.

The front side of the VOPcF_8_ HS shows the π–π interaction between benzene moieties of neighboring molecules with the angle/distance/shift values of 4.454°/3.424 Å/1.123 Å, while the back surface is characterized by π–π interactions between a benzene moiety of one molecule and a pyrrole fragment of another molecule with angle/distance/shift values of 3.995°/3.228 Å/1.068 Å.

Thus, the introduction of eight F-substituents has different effects on the phthalocyanines of various metals. In the case of CoPcF_8_, this effect is negligible and CoPcF_8_ remains isostructural with its tetrafluoro-substituted analogue CoPcF_4_-p [13]. However, in the case of ZnPcF_8_, this leads to a complete change in the packing motif from conventional stacks to 2D molecular layers formed by staggered molecular stacks, which reduces the number of π–π interactions between neighboring molecules compared to ZnPcF_4_-p [44]. VOPcF_8_ molecules are packed into interconnected molecular chains, which can be considered as a cross between the molecular chains in the triclinic polymorph of VOPc-II and 2D molecular layers in VOPcF_16_. Although the nature of the π–π interactions between neighboring molecules of VOPcF_8_ does not differ much from other fluorinated vanadyl phthalocyanines, the same cannot be said about close F...F and F...H contacts. Compared to VOPcF_4_-p, F...F close contacts in VOPcF_8_ are much less noticeable, which may lead to better crystallinity and lower micro stresses in the crystal lattice.

### 2.3. Vibrational Spectra of MPcF_8_

**Figure 6:**
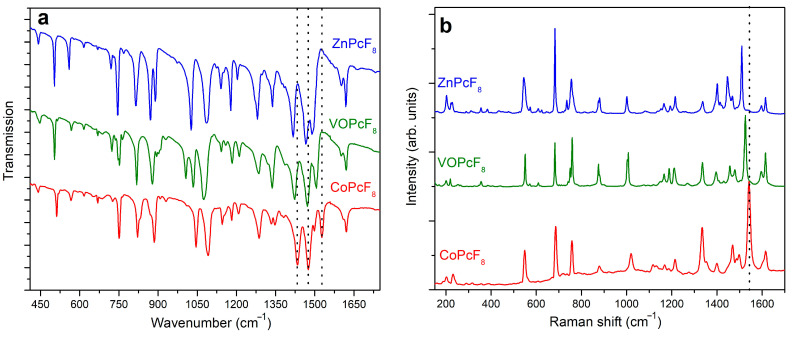
IR (**a**) and Raman (**b**) spectra of MPcF_8_ (M=Co, VO, Zn).

IR and Raman spectra of MPcF_8_ powders are shown in Figure 6a,b. The bands in the range from 1350 to 1620 cm^−1^, which are assigned mostly to C=C and C=N stretching vibrations, are similar to those of tetrafluoro-substituted derivatives [45], while there is a noticeable difference in the position and intensities of bands with a significant contribution of C-F, C_β_-C_γ_-F, and C_β_-C_γ_-H vibrations in the range from 900 to 1350 cm^−1^.

The introduction of more F-substituents into the phthalocyanine macrocycle leads also to alterations in the range of 600–900 cm^−1^ in both IR and Raman spectra in comparison with MPcF_4_ derivatives. The bands in this region corresponding mostly to M-N_α_ stretching vibrations and macroring deformations are mixed with C-C-H deformations and C-F stretching [45]. An increase in the number of withdrawing F-substituents and, as a consequence, a decrease in the number of C-C-H fragments leads to a change in the intensity and the form of vibrations in this region.

It is known that the range from 1350 to 1550 cm^−1^ in the vibrational spectra of metal phthalocyanines is considered as a “marker” of central metals. Similar to unsubstituted, tetrafluorinated, and perfluorinated MPc [45,46,47,48], MPcF_8_ exhibits bands dependent on central metals in this spectral region. For example, the band at 1528 cm^−1^ in the IR spectrum of CoPcF_8_ shifts to 1506 and 1492 cm^−1^ when passing to VOPcF_8_ and ZnPcF_8_, respectively (Figure 6a). The bands at 1434 cm^−1^ in the IR spectrum of CoPcF_8_ are also observed at lower wavenumbers in the spectra of VOPcF_8_ and ZnPcF_8_. In the Raman spectra, the band, which is most sensitive to the central metal, lies at 1542 cm^−1^ for CoPcF_8_ and shifts to 1526 cm^−1^ and 1510 cm^−1^ in the spectra of VOPcF_8_ and ZnPcF_8_, respectively (Figure 6b). According to the DFT calculations carried out in previous works [45,46,47,48], these bands are assigned to the displacements of C_α_-N_β_-C_α_ bridges between isoindole groups in the MPc macrocycle and the change of the cavity size during the macrocycle vibration. For this reason, the shift of these bands is in good correlation with the cavity size (more precisely, with the N_α_-M-N_α_ distance) in the phthalocyanine molecules. Indeed, similarly to other phthalocyanine derivatives, the experimental N_α_-M-N_α_ distance in MPcF_8_ increases in the order of Co (3.83 Å) > VO (3.91 Å) > Zn (3.97 Å).

### 2.4. Study of MPcF_8_ Powders and Thin Films by XRD and UV-vis Spectroscopy

**Figure 7:**
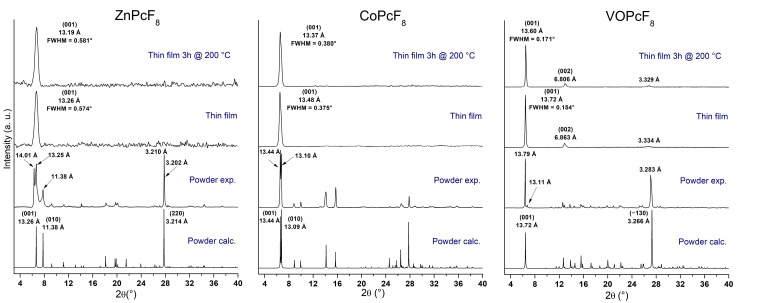
XRD patterns for MPcF_8_ powders (experimental and calculated) and thin films before and after heating for 3 h at 200 °C.

XRD patterns for bulk powders and as-deposited thin films of MPcF_8_ are shown in Figure 7. The XRD patterns of the films heated at 200 °C for 3 h in air are also given because it is known that films of some metal phthalocyanines undergo a phase transition when heated [49,50].

Polycrystalline MPcF_8_ powders were obtained by gradient sublimation in a vacuum. Among the three investigated phthalocyanines, only the CoPcF_8_ powder pattern completely coincides with the calculated one. Two small diffraction peaks with d = 13.11 Å and d = 8.317 Å on the XRD pattern of VOPcF_8_ powder do not coincide with the calculated powder pattern and belong to another crystalline phase of VOPcF_8_, which is present in an insignificant amount in the investigated powder.

ZnPcF_8_ powder contains two crystal phases mixed in a comparable amount. The structural data of the first phase are presented in Table 1. We also tried to isolate single crystals of the second phase of ZnPcF_8_ but only one small (less than 30 μm in length and 5 μm in thickness) defective needle crystal, which did not show any diffraction spots with a resolution better than 3 Å on the detector frame with a collection time of 300 s/frame, was found. For this reason, only its unit cell parameters (measured by ~100 collected reflections) were determined: a = 3.655(7) Å; b = 13.38(2) Å; c = 13.67(2) Å; α = 85.21(4)°; β = 88.52(9)°; γ = 82.36(6)°; and V = 660(3) Å^3^. According to the unit cell parameters, the second phase of ZnPcF_8_ is isostructural with CoPcF_8_ and CuPcF_8_.

A comparison of XRD patterns of thin films with the calculated diffractograms shows that all three films consist of a single crystal phase, which corresponds to the crystal structure data. Only one strong diffraction peak corresponding to the plane (001) is observed on the diffraction pattern of each film, which indicates a strong preferred orientation with the vast majority of crystallites oriented with a (001) crystallographic plane parallel to the substrate surface. Two additional weak diffraction peaks are observed on the diffractogram of the VOPcF_8_ film; the peak with d = 6.863 Å corresponds to the plane (002), while the peak at 3.334 Å does not exactly coincide with the plane (004), which has d = 3.432 Å. This may be due to the fact that the preferred orientation of the VOPcF_8_ film is not ideal, and the peak at 3.334 Å is actually a mixture of (004) and (−130) peaks, which are noticeable in the powder diffraction pattern.

**Figure 8:**
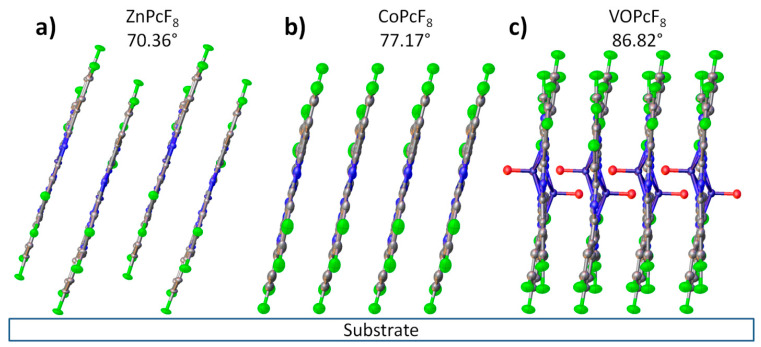
Orientation of ZnPcF_8_ (**a**), CoPcF_8_ (**b**), and VOPcF_8_ (**c**) molecules in the thin films relative to the substrate surface.

Since all MPcF_8_ thin films have strong a preferred orientation with a known plane of preferred orientation, it is possible to calculate the angle between molecules and the substrate surface using the data on the structure of single crystals. The angle values are 70.36° for ZnPcF_8_, 77.17° for CoPcF_8_, and 86.82° for VOPcF_8_. The scheme of the orientation of MPcF_8_ molecules in the thin films relative to the substrate surface is shown in Figure 8.

**Figure 9:**
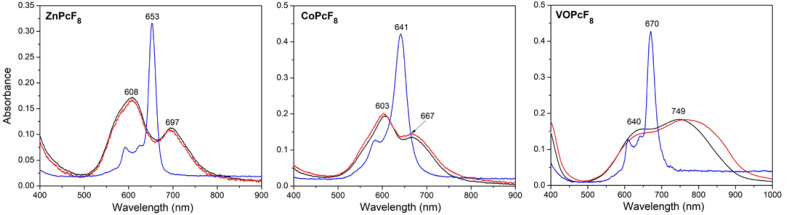
Optical absorption spectra of MPcF_8_ (M=Zn, Co, VO) solutions in THF (blue lines) and films deposited on glass slides before (black lines) and after heating at 200 °C for 3 h (red lines).

UV-vis spectra of the films are given in Figure 9 in comparison with the spectra of MPcF_8_ solutions in THF. All spectra contain a typical Q-band, which is attributed to the electron transitions from HOMO a_1u_ to the LUMO e_g_ and is very sensitive to the surrounding of an MPc molecule and intermolecular interactions [51,52].

The maxima of the Q-bands in the UV-vis spectra of CoPcF_8_ and ZnPcF_8_ solutions are located at 641 and 653 nm, respectively. The Q-band in the spectrum of a CoPcF_8_ film deposited on a glass slide is split into two components Q_I_ and Q_II_ with maxima at 603 and 667 nm. This spectrum is similar to those for CoPcF_4_ [53] and α-CoPc [54]. The Q-band splitting is due to intermolecular interaction and different arrangements of molecules relative to each other in a unit cell. According to the XRD data, CoPcF_8_ is isostructural with them. The UV-vis spectrum of ZnPcF_8_ also contains two Q-band components; however, the difference between their wavelengths is noticeably larger: Q_I_ at 608 nm and Q_II_ at 697 nm. The packing of molecules in ZnPcF_8_ differs from that in CoPcF_8_. As it was shown above, the ZnPcF_8_ molecules are staggered inside the stack. The UV-vis spectrum of a VOPcF_8_ film exhibits a broad red-shifted Q-band, which is similar to that in the spectra of non-planar VOPc and TiOPc derivatives [55] as well as VOPcF_4_ [23] and is a characteristic of J-aggregate formation [56].

Annealing in the air at 200 °C for 3 h had no significant effect on thin films. The observed diffraction peaks are slightly shifted to the right (interplanar values become smaller), which is caused by the release of mechanical stress in thin films. Assuming that the instrumental peak broadening is 0.05° (measured from an SRM−660a LaB_6_ powder sample), the size of the coherent scattering region for each film was estimated using the Scherrer equation and equal to 16.9 nm for ZnPcF_8_, 85 nm for CoPcF_8_, and 27.2 nm for VOPcF_8_. After annealing, these values became 16.6 nm, 73 nm, and 26.8 nm, respectively. The angles between phthalocyanine molecules (least squares plane through all atoms except hydrogen, oxygen, and vanadium) and the substrate surface, determined on the basis of the crystal structure data, were 70.36° for ZnPcF_8_, 75.45° for CoPcF_8_, and 86.82° for VOPcF_8_. The UV-vis spectra of CoPcF_8_ and ZnPcF_8_ films after heating also did not undergo noticeable changes. In the UV-vis spectrum of VOPcF_8_, the Q-band only became broader.

### 2.5. Study of The Conductivity of MPcF_8_ Films

**Figure 10:**
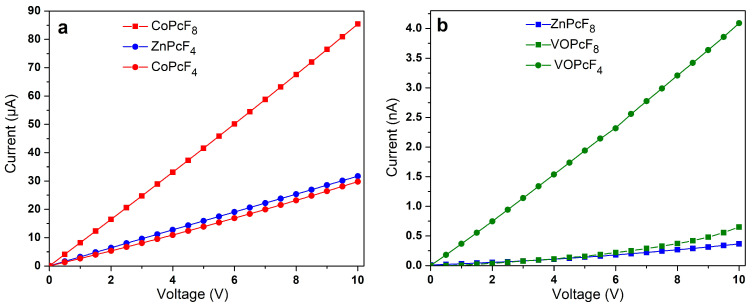
*I(V)* dependencies of CoPcF_8_ (**a**) and ZnPcF_8_, VOPcF_8_ (**b**) thin films in comparison with their tetrafluoro-substituted analogues CoPcF_4_-p, ZnPcF_4_-p (**a**) and VOPcF_4_-p (**b**).

To investigate the effect of the central metal ion on conductivity, the I(V) dependencies of as-deposited MPcF_8_ (M=Zn(II), Co(II), VO) films were measured using a Keithley 236 electrometer (Figure 10a,b). For this purpose, the films were deposited on glass substrates with interdigitated Pt electrodes. A CoPcF_8_ film demonstrates ohmic conductivity in the investigated range from 0 to 10 V. The I(V) dependencies of ZnPcF_8_ and VOPcF_8_ are linear below 3 V and 2.5 V, respectively, while the shape of curves changes above these values with the slopes of a log*I* versus log*V* at about 1.3 for ZnPcF_8_ and 1.7 for VOPcF_8_. These values are below *n* = 2 in the dependence of $I\propto V^{n}$, which is typical for the space-charge-limited conduction (SCL) mechanism [57]. In the case of the investigated MPcF_8_ (M=Zn(II), Co(II), VO) films, the ohmic conduction mechanism dominated the SCL conduction. The lateral d.c. conductivity (*σ*_//_) estimated in the linear range was close for the films of ZnPcF_8_ (1.9·10^−9^ Ω^−1^m^−1^) and VOPcF_8_ (1.3·10^−9^ Ω^−1^m^−1^). The *σ*_//_ of CoPcF_8_ was noticeably higher and equal to 6.2·10^−4^ Ω^−1^m^−1^. The conductivity of MPcF_8_ films was also compared with that of their tetrafluorinated analogues (Figure 10a,b).

The conductivity and charge-carrier mobility of polycrystalline MPc films are determined both by the packing of phthalocyanine molecules in the crystallite and by the morphology of the film. Jiang with co-authors [37,58] studied the dependence of the charge-carrier mobility in MPc single crystals on the distances between neighboring molecules along the shortest axis with the closest parallel π-stackings. It was shown that as the intermolecular distances along their shortest packing axis in unsubstituted H_2_Pc and MPcs decreased, the hole mobility gradually increased. This approach is reasonable in the case of isostructural phthalocyanines. CoPcF_8_ and ZnPcF_8_ investigated in this work have different structures; the distances between neighboring molecules in stacks are very close but the packing motifs are different, which leads to the different π–π interactions between neighboring molecules in the stack. It was shown above using HS analysis that in the case of ZnPcF_8_ only pyrrole moieties participate in one π–π interaction, while in the case of CoPcF_8_ the whole molecule is involved in π–π interaction with neighboring molecules in the stack (Figure 5). Such a better overlap of π-orbitals may contribute to an increase in the conductivity of CoPcF_8_ films. Moreover, according to the XRD analysis, there are no disordered atoms in the structure of CoPcF_8_, which leads to better crystallinity of the films because the fewer defects and microstresses in the crystal lattice, the better the crystallinity of the films. This fact in turn may lead to the better conductivity of CoPcF_8_ films. The same character of interactions as in CoPcF_8_ was also observed in ZnPcF_4_-p and CoPcF_4_-p [13]. The *σ*_//_ values of the films of these phthalocyanines were comparable with the conductivity of CoPcF_8_ films and equal to 2.1·10^−4^ and 2.6·10^−4^ Ω^−1^m^−1^ for ZnPcF_4_-p and CoPcF_4_-p, respectively.

Vanadyl phthalocyanine molecules have a different packaging style due to their non-planar structure. HS of VOPcF_8_ (Figure 5) showed that four benzene rings of each molecule form two pairs of π–π bonds with two adjacent molecules, making molecular chains. Comparison of HS shows that VOPcF_4_ (2.9·10^−8^ Ω^−1^m^−1^) has more π–π contacts than VOPcF_8_, including contacts between benzene fragments and between the benzene ring and macrocycle of the adjacent molecule [13].

## 3. Materials and Methods

ZnPcF_8_, CoPcF_8_, and VOPcF_8_ were synthesized by template synthesis [59] by fusing a mixture (4:1) of difluorophthalonitrile (abcr, Karlsruhe, Germany, CAS 134450−56−9) with salts of the corresponding metals, viz., zinc acetate dihydrate (Sigma-Aldrich, Saint Louis, MO, USA, CAS 5970−45−6), CoCl_2_ 391 (Sigma-Aldrich, Saint Louis, MO, USA, CAS 7746−79−9), or VCl_3_ (Sigma-Aldrich, Saint Louis, MO, USA, CAS 7718−98−1), respectively. Difluorophthalonitrile and the corresponding amount (4:1 molar ratio) of a metal salt were ground in a mortar and placed in an ampoule. The mixture was heated at 190–210 °C for several hours, then the resulting dark blue or dark green solid product was crushed and placed in a new clean ampoule for subsequent purification by sublimation. The resulting powders were purified twice by sublimation in a vacuum (10^−5^ Torr); the yield after sublimation was around 50%.

As a result of sublimation, single crystals of ZnPcF_8_, CoPcF_8_, and VOPcF_8_ were obtained.

ZnPcF_8_: C_32_H_8_F_8_N_8_Zn. Anal. Calc: C, 53.2; H, 1.1; N, 15.5; F, 21.1%. Found: C, 53.4; H, 1.3; N, 15.5; F, 21.0%. IR spectrum (KBr; ω, cm−1): 1618; 1603; 1489; 1465; 1339; 1281; 1204; 1178; 1140; 1086; 1026; 889; 870; 814; 746; 615; 559; 503; 441.

CoPcF_8_: C_32_H_8_F_8_N_8_Co. Anal. Calc: C, 53.7; H, 1.1; N, 15.7; F, 21.2%. Found: C, 53.8; H, 1.1; N, 15.9; F, 21.4%. IR spectrum (KBr; ω, cm^−1^): 1620; 1601; 1506; 1472; 1423; 1337; 1286; 1211; 1184; 1142; 1074; 1034; 895; 878; 818; 752; 688; 615; 567; 503; 447.

VOPcF_8_: C_32_H_8_F_8_N_8_VO. Anal. Calc: C, 53.1; H, 1.1; N, 15.5; F, 21.0%. Found: C, 53.3; H, 1.2; N, 15.6; F, 21.1%. IR spectrum (KBr; ω, cm^−1^): 1620; 1609; 1528; 1499; 1475; 1433; 1347; 1287; 1208; 1182; 1146; 1092; 1045; 930; 885; 870; 822; 750; 669; 617; 567; 511; 442.

Thin films of ZnPcF_8_, CoPcF_8_, and VOPcF_8_ were deposited by an OMBD technique at a residual pressure of 10^−5^ Torr and evaporation temperature of 450–460 °C. Glass slides were used as substrates. The films’ thicknesses were determined by the method of spectral ellipsometry using a spectroscopic ellipsometer ELLIPS 1771 SA (ISP, Novosibirsk, Russia) [60] as described in our previous work [61]; thicknesses were determined to be 85, 80, and 87 nm for ZnPcF_8_, CoPcF_8_, and VOPcF_8_ films, respectively.

IR and Raman spectra were recorded with a Vertex 80 FTIR spectrometer (Ettlingen, Germany) and a LabRAM Horiba single spectrometer (Montpellier, France) (488 nm line of an Ar+ laser), respectively.

Crystal structure data were obtained using a single-crystal diffractometer Bruker X8 (sealed Mo-anode tube with a graphite monochromator, 4-circle goniometer, APEX II CCD detector) and Venture single-crystal diffractometer Bruker D8 (Billerica, MA, USA) (Incoatec IμS 3.0 microfocus X-ray source with Mo-anode, 3-circle goniometer, PHOTON III C14 CPAD detector). Both diffractometers were equipped with Oxford Cryosystems Cryostream 800 (Oxford, United Kingdom) plus open-flow nitrogen coolers, which were used to maintain the sample temperature at 150(±2) K. The data acquisition strategy consisted of several ω-scans with 0.5° wide frames. The raw data frame collection, data reduction, absorption correction, and global unit cell refinement were performed in the APEX3 V2018.7–2 software package (SAINT 8.38A, SADABS−2016/2) (Madison, Wisconsin, USA) [62]. The resulting hkl datasets were processed in Olex2 v.1.5 10 (Durham, United Kingdom) [63] using SHELXT 2018/2 [64] and SHELXL 2018/3 [65] (Göttingen, Germany) for the structure solution and refinement. ZnPcF_8_, CoPcF_8_, and VOPcF_8_ structural data were deposited to The Cambridge Crystallographic Data Centre (CCDC) with the numbers 2177575, 2177576, and 2177577 and can be obtained from https://www.ccdc.cam.ac.uk/structures/ (accessed on 5 July 2022).

Powder and thin film diffraction patterns were obtained in the 2θ range of 3–40° using a Bruker D8 (Billerica, MA, USA) advance powder diffractometer (vertical θ-θ goniometer in the Bragg-Brentano geometry, Cu-anode sealed tube without a β-filter 40mA @ 40 kV, LYNXEYE XE-T compound silicon strip detector, motorized divergence slit), with a step of 0.01023°, an acquisition time of 2 s/step, and a fixed sample illumination area.

Electronic absorption spectra of MPcF8 solutions and films were recorded using a UV–Vis−3101PC ‘‘Shimadzu” (Kyoto, Japan) scanning spectrophotometer.

I(V) dependencies of MPcF_8_ films were measured using a Keithley 236 electrometer. The films were deposited onto glass slides with Pt IDE (Metrohm, DropSens, Spain, the dimension of the gaps was 10 μm; the number of digits was 125 × 2 with a digit length equal to 6760 μm).

## 4. Conclusions

In this work, octafluoro-substituted phthalocyanines of zinc, vanadyl, and cobalt (MPcF_8_, M=Zn(II), Co(II), VO) were synthesized and studied. The structures of CoPcF_8_ and VOPcF_8_ single crystals were determined for the first time. CoPcF_8_ crystallizes in a triclinic system with Z=1 and a space group P−1 and is isostructural to α-CoPc and previously reported CoPcF_4_-p and CuPcF_8_. VOPcF_8_ also crystallizes in a P−1 space group but with Z=2. Judging from the unit cell parameters, this minor phase of ZnPcF_8_ is isostructural to CoPcF_8_ and CuPcF_8_.

It was shown that the presence of eight peripheral F-substituents has a different effect on molecular packing and intermolecular contacts in different MPcF_8_. In the case of CoPcF_8_, no significant changes were observed compared to α-CoPc and CoPcF_4_-p. ZnPcF_8_ molecules are packed in a staggered manner, forming two-dimensional molecular layers, usually observed in non-planar phthalocyanines. The number of π–π interactions between ZnPcF_8_ molecules decreased compared to ZnPcF_4_-p. VOPcF_8_ molecules are packed in molecular chains that are connected to each other along the *b* axis. Compared to VOPcF_4_-p, close F…F and F…H contacts between VOPcF_8_ molecules are much weaker.

All three studied MPcF_8_ formed highly oriented single-phase thin films when deposited onto glass substrates using a PVD method. Their crystal phases correspond to the crystal structure of single crystals. The angles between the macrocycle plane and the substrate surface were 70.36° for ZnPcF_8_, 77.17° for CoPcF_8_, and 86.82° for VOPcF_8_.

## Figures and Tables

**Table 1:** Unit cell parameters and refinement statistics for MPcF_8_.

Compound	ZnPcF_8_	CoPcF_8_	VOPcF_8_
Empirical formula	C_32_H_8_F_8_N_8_Zn	C_32_H_8_F_8_N_8_Co	C_32_H_8_F_8_N_8_OV
Formula weight	721.83	715.39	723.40
Temperature/K	150	150	150
Crystal system	triclinic	triclinic	triclini
Space group	P−1	P−1	P−1
a/Å	8.2156(5)	3.6061(2)	9.0821(5)
b/Å	11.5255(7)	13.0280(7)	10.3447(5)
c/Å	14.0379(8)	13.4901(7)	13.9293(8)
α/°	78.693(2)	84.097(2)	98.578(2)
β/°	73.840(2)	89.055(2)	92.165(2)
γ/°	89.047(2)	82.976(2)	92.909(2)
Volume/Å^3^	1250.92(13)	625.67(6)	1291.01(12)
Z	2	1	2
ρ_calc_g/cm^3^	1.916	1.899	1.861
μ/mm^−1^	1.087	0.791	0.492
Radiation	MoKα (λ = 0.71073)	MoKα (λ = 0.71073)	MoKα (λ = 0.71073)
2Θ range for data collection/°	3.082 to 61.038	3.036 to 51.362	4.594 to 56.68
Reflections collected	15,790	9899	20,656
Independent reflections	7629 [R_int_ = 0.0271, R_sigma_ = 0.0399]	2377 [R_int_ = 0.0489, R_sigma_ = 0.0476]	6423 [R_int_ = 0.0375, R_sigma_ = 0.0443]
Data/restraints/parameters	7629/0/442	2377/0/223	6423/0/451
Goodness-of-fit on F^2^	1.030	1.063	1.039
Final R indexes [I > = 2σ (I)]	R_1_ = 0.0354, wR_2_ = 0.0886	R_1_ = 0.0539, wR_2_ = 0.1314	R_1_ = 0.0518, wR_2_ = 0.1393
Final R indexes [all data]	R_1_ = 0.0551, wR_2_ = 0.0978	R_1_ = 0.0760, wR_2_ = 0.1427	R_1_ = 0.0762, wR_2_ = 0.1552
Largest diff. peak/hole/e Å^−3^	0.50/−0.35	1.05/−0.36	1.22/−0.39
Number in CCDC	2177575	2177576	2177577

**Table 2:** Intermolecular and interatomic distances in MPcF_8_.

Distances	ZnPcF_8_	CoPcF_8_	VOPcF_8_
between layers, Å	3.173	N/A	3.382
between molecules, Å	3.217	3.299	3.411/3.354
d_norm_ (min/max), Å	−0.1495/1.3175	−0.1985/1.3833	−0.1477/1.4484
metal…metal, Å	4.867	3.606	5.871
C…C/C…N close contacts, Å	3.211−3.260 < 3.235>	3.271−3.275 < 3.273>	3.204−3.352 < 3.288>
O…H close contacts, Å	N/A	N/A	2.409−2.505 < 2.457>
F…H close contacts, Å	2.354−2.401 < 2.368>	2.440	2.404−2.509 < 2.451>
F…F close contacts, Å	N/A	N/A	2.864
π…π interactions (angle/distance/shift)	2.603°/3.232 Å/0.777 Å 1.219°/3.230 Å/0.786 Å	0°/3.251−3.357 Å/1.318−1.561 Å	4.454°/3.424 Å/1.123 Å 3.995°/3.228 Å/1.068 Å

## Data Availability

The data presented in this study are available on request from the corresponding author. ZnPcF_8_, CoPcF_8_, and VOPcF_8_ structural data can be obtained from https://www.ccdc.cam.ac.uk/structures/ (accessed on 5 July 2022) (numbers 2177575, 2177576, and 2177577).
